# Secure Angle-Based Geometric Elimination (SAGE) for Microrobot Path Planning

**DOI:** 10.3390/mi16111273

**Published:** 2025-11-12

**Authors:** Youngji Ko, Seung-hyun Im, Hana Choi, Byungjeon Kang, Jayoung Kim, Taeksu Lee, Jong-Oh Park, Doyeon Bang

**Affiliations:** 1 Department of AI Convergence, Chonnam National University, 77 Yongbong-ro, Buk-gu, Gwangju 61186, Republic of Korea; 200684@kimiro.re.kr (Y.K.); tmdguszza@kimiro.re.kr (S.-h.I.); bjkang8204@jnu.ac.kr (B.K.); 2 Robot Research Initiative, Chonnam National University, 77 Yongbong-ro, Buk-gu, Gwangju 61186, Republic of Korea; 3 Korea Institute of Medical Microrobotics, 43-26, Cheomdangwagi-ro 208-beon-gil, Buk-gu, Gwangju 61011, Republic of Korea; gua05061@kimiro.re.kr (H.C.); jaya@cbnu.ac.kr (J.K.); tslee@kimiro.re.kr (T.L.); 4 Graduate School of Data Science, Chonnam National University, 77 Yongbong-ro, Buk-gu, Gwangju 61186, Republic of Korea; 5 Department of Biosystems Engineering, Chungbuk National University, Cheongju 28644, Republic of Korea

**Keywords:** microrobot, navigation, path simplification, collision avoidance, lab-on-a-chip, lab automation

## Abstract

Microrobot navigation in constrained environments requires path planning methods that ensure both efficiency and collision avoidance. Conventional approaches, which typically combine graph-based path finding with geometric path simplification, effectively reduce path complexity but often generate collision-prone paths because wall boundaries are not considered during simplification. Therefore, to overcome this limitation, we present Secure Angle-based Geometric Elimination (SAGE), a single-pass path-simplification algorithm that converts pixel-level shortest paths into low-complexity trajectories suitable for real-time collision-free navigation of microrobots. SAGE inspects consecutive triplets (*p_i_*, *p_i+_*_1_, *p_i+_*_2_) and removes the middle point when the turning angle is smaller than threshold (∠*p_i_p_i+_*_1_*p_i+_*_2_ ≤ *θ_th_*) or the direct segment (*p_i_* → *p_i+_*_2_) is collision-free. Quantitative analysis shows that SAGE achieves approximately 5% shorter path length, 20% lower turning cost and 0% collision rate, while maintaining computation comparable to the Ramer–Douglas–Peucker algorithm. Integration with Dijkstra and RRT planners confirms scalability across complex maze and vascular environments. Experimental microrobot demonstrations show navigation with complete collision avoidance, establishing SAGE as an efficient and reliable framework for high-speed microrobot navigation and automation in lab-on-a-chip, chemical-reaction and molecular-diagnostic systems.

## 1. Introduction

Microrobots are attracting significant attention, recognized for their advantages in noninvasive treatment and diagnosis, as well as the efficiency of drug delivery. Microrobots can increase drug delivery efficiency by delivering drugs directly to the target, thereby reducing drug usage and side effects from drug overuse [1,2,3]. Beyond disease treatment, microrobots are also being used in in vitro diagnostics and chemical reactions [4]. Furthermore, automation using AI is being widely adopted in many fields. Research is also underway to incorporate AI into microrobots [5,6]. In particular, the introduction of microrobots and AI in the fields of in vitro diagnostics and chemical reactions can protect workers from potential hazards [7]. Since the primary role of microrobots is to deliver substances, such as drugs, to specific targets, automating this process through the introduction of AI can maximize efficiency in areas like targeted therapy, diagnosis, and chemical synthesis.

Microfluidic systems used in in vitro diagnostics and chemical reactions have evolved to perform complex tasks, including exosome isolation, concentration, and even mechanical disruption for molecular analysis [8,9,10,11,12]. Despite these advances, most existing platforms rely on passive or externally driven fluid motion, lacking the capability for active and adaptive droplet transport within confined channels. This limitation restricts precise spatiotemporal control over reagent mixing, reaction sequencing, and on-chip automation. Therefore, microrobotic approaches capable of autonomous droplet navigation and dynamic operation in microscale environments are required.

However, microrobots operate in environments both inside and outside the body, where paths are narrow and numerous obstacles are likely to be encountered. Therefore, collisions with obstacles or walls must be considered when carrying materials and moving robots. Collisions can not only cause damage to the mounted objects and robots, but can also cause fatal risks to the patient, such as bleeding, and when multiple microrobots move together, collisions can lead to unintended interactions between the robots [13].

Many path planning algorithms currently under development have been implemented for autonomous vehicles, including automobiles, drones, indoor/outdoor robots and microrobots [14,15,16,17,18]. Liu, Y. et al. proposed the safety-enhanced navigation planning (SENP) algorithm to generate safe, smooth, and short-distance paths in complex channels or narrow corridor such as blood vessels [19]. This study introduced the safe artificial potential field (SAPF) to resolve the oscillation problem that occurs in narrow corridor, and demonstrated a faster convergence speed than the conventional RRT* method by incorporating a goal-bias strategy. This method is related to path generation algorithms rather than path simplification algorithms. Additionally, this research team proposed CMA-ES-RRT, a path planning approach that first quickly generates an initial path using RRT and then introduces covariance matrix adaptation evolution strategy (CMA-ES) to smooth and shorten the path [20]. This post-processing path planning method showed results where the total path length was reduced and the total change in angle was decreased compared to the original path. Chehelgami, S. et al. proposed a global path planning method capable of generating collision-free, safe paths even in complex environments through deep learning [21]. They introduced a new loss function, the Mean Squared Error–Nearest Edge Repulsive (MSE-NER), which allows for securing a safe margin from obstacles, and demonstrated that paths could be generated faster and with a higher success rate than existing methods.

It was determined that there is a need for an algorithm that is faster at path simplification, guarantees a 100% success rate, and is completely collision-free compared to existing studies. Therefore, we propose a fast, safe, and versatile path simplification algorithm. This algorithm is designed to integrate seamlessly with various path planning algorithm, including but not limited to the RRT algorithm—whose probabilistic nature inherently limits the guarantee of a 100% success rate. By ensuring complete collision-free, this approach directly facilitates the successful deployment of microrobots in applications such as in vitro diagnostics and chemical synthesis. We targeted static, confined, lab-on-a-chip environments with stable overhead imaging and a pre-registered binary map. Pixel-level planning becomes practical under these conditions, because obstacle inflation by morphological erosion (≈½ robot diameter) guarantees a conservative safety buffer. SAGE then simplifies the pixel path only when a shortcut segment is verified as collision-free on this inflated map, preserving safety while reducing turns.

A variety of path planning algorithms are currently in common use, including graph-based methods like the Dijkstra algorithm and the RRT algorithm. There are also modified or improved versions, such as the RRT* algorithm. The RRT algorithm works by randomly selecting the next node within the entire search space to expand a tree, generating a path to the goal. It repeats this process a specified number of times and selects the best path from the results. Because the RRT algorithm does not guarantee the shortest path, the RRT* algorithm was proposed as an improvement [22]. Tests on the RRT* algorithm showed that a higher number of iterations leads to longer path generation times, and the resulting path is not consistently the same. Experiments also confirmed that because the algorithm randomly selects the next node, there are instances where path generation fails. Considering the limitations of RRT and RRT* algorithms, this study adopts the Dijkstra algorithm, which can generate a consistent path with high speed every time. The Dijkstra algorithm finds the shortest path by using the positive weight values assigned to each node to create an optimal path with the minimum cost [23]. However, a path generated in this manner can include an excessive number of pixel-based points and unnecessary detours on a binary grayscale image, which slows down robot movement. Therefore, an algorithm is needed to simplify the generated path.

**Figure 1:**
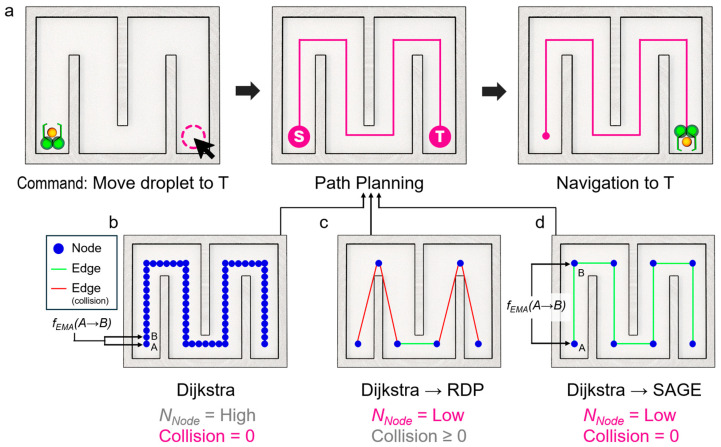
Overall concept of microrobot-based path planning and simplification. (**a**) Schematic illustration of a microrobot-assisted automated task, such as microdroplet delivery to a target location. (**b**) Path generation using a base searching algorithm (e.g., BFS or Dijkstra), which yields the shortest path but results in a large number of turns and path segments, thereby causing low navigation speed. (**c**) Path simplification using the Ramer–Douglas–Peucker (RDP) algorithm after base searching. This reduces the number of path segments and turns, leading to faster navigation. However, since RDP does not consider wall boundaries, the simplified path may cause collisions. (**d**) Path simplification using the proposed Secure Angle-based Geometric Elimination (SAGE) algorithm after base searching. SAGE reduces the number of turns and segments while incorporating angle-based collision elimination, thereby enabling faster and collision-free navigation.

The Ramer–Douglas–Peucker (RDP) algorithm is one of the most commonly used path simplification algorithms. However, a significant drawback is that it simplifies the path based on the point farthest from a line segment, which completely disregards potential collisions with obstacles [24]. This makes it unsuitable for a robot that must safely perform a defined mission. To overcome the limitations of the RDP algorithm, this study proposes a path simplification method that uses angles to remove unnecessary points (Figure 1). At the same time, it utilizes the Bresenham algorithm to consider and avoid collisions with obstacles. We will validate the performance of our proposed algorithm by comparing it to the RDP algorithm and proving its superiority through real-time experiments with a physical robot.

## 2. Materials and Methods

### 2.1. Materials

The computer specifications used to write and run the code, conduct the experiments, and control the microrobot actuation system are as follows: Windows 11 (Microsoft, Redmond, WA, USA), AMD Ryzen 9 8940HX with Radeon Graphics, 32.0 GB RAM (Advanced Micro Devices, Inc., Santa Clara, CA, USA). Python and library versions are as follows: Python 3.12.11, OpenCV 4.12.0, Numpy 2.0.1, Matplotlib 3.10.0.

### 2.2. Methods

#### 2.2.1. Secure Angle-Based Geometric Elimination (SAGE) Algorithm

The Secure Angle-based Geometric Elimination (SAGE) Algorithm is a path simplification algorithm proposed for real-time in vitro diagnostics and chemical reactions, used to post-process paths generated by a planning algorithm. A path produced by a typical path planning algorithm on a binary grayscale image is a sequence of continuous pixels. As robot actuation data is derived from pixel information, a high pixel count directly increases both the information transmission time and the robot navigation time. Consequently, a path simplification algorithm is required to minimize the number of pixels while ensuring the path remains collision-free.

**Figure 2:**
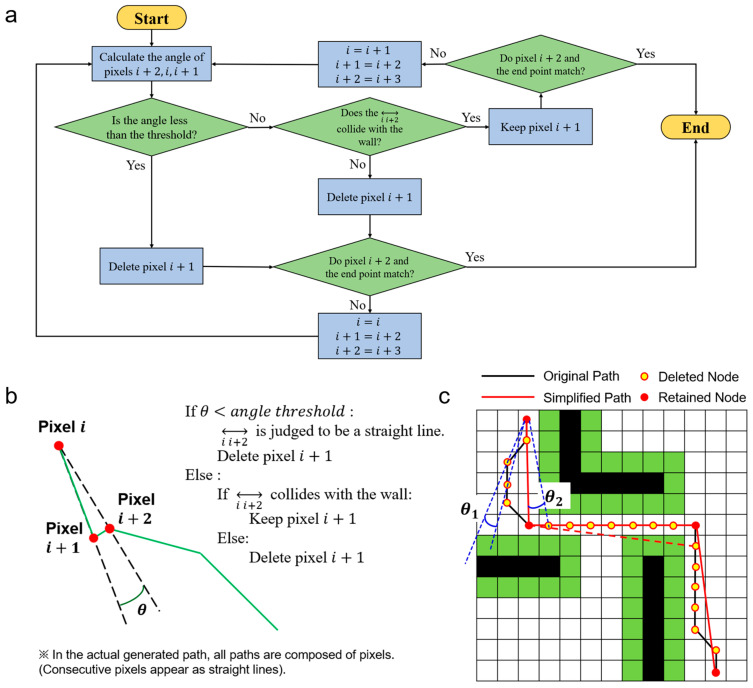
Principle and implementation of the proposed Secure Angle-based Geometric Elimination (SAGE) algorithm. (**a**) Flowchart of the SAGE procedure illustrating the iterative process of angle evaluation, collision checking and pixel elimination until the endpoint is reached. (**b**) Schematic illustration of the angle-based simplification process. For each triplet of consecutive pixels, the turning angle (*θ*) is compared with a predefined threshold. If *θ* is below the threshold or the direct line segment between pixels *i* and *i +* 2 is collision-free, the intermediate pixel (*i +* 1) is removed. (**c**) Example of path simplification using the SAGE algorithm, demonstrating reduced path complexity and collision-free trajectory generation.

SAGE achieves both high speed and a guaranteed collision free path simplification by leveraging the inherent safety of the base path. A path generated by a graph-search algorithm like Dijkstra is, by its nature, collision-free. This is because the algorithm operates on a graph where the nodes and edges are drawn exclusively from the valid, free-space (non-obstacle) regions of the map. Therefore, any path it finds is guaranteed to consist only of safe points. SAGE’s task is to simplify this already-safe path. It iterates through consecutive triplets of points (current pixel (*i*), next pixel (*i +* 1), and pixel (*i +* 1)) and calculates the angle *θ* between them (Figure 2a). This angle is used to distinguish between two scenarios (straight and curved section).

- If the calculated angle is less than or equal to a small angle threshold, SAGE identifies this as a straight-line segment of the already-safe path. The intermediate pixel (*i +* 1) is rapidly removed without a collision check. This action is inherently safe, as simplifying a straight line that already exists in a free-space region cannot create a collision. This step ensures SAGE’s high computational speed.
- Conversely, if the calculated angle is greater than the threshold, SAGE treats this route as a potential shortcut, which needs collision verification. If this new shortcut is found to be unsafe at any point, the simplification is rejected, and the original, safe intermediate point (*i +* 1) is kept.

To determine the presence of a collision, Bresenham’s line algorithm was adopted. Bresenham’s line algorithm is a method for drawing lines in computer graphics using only integer coordinates. It works by incrementally increasing the *x*-coordinate or *y*-coordinate by one pixel from the starting point to the endpoint and deciding whether to increment or maintain the other coordinate. This method is highly efficient because it can be programmed without using multiplication or division [25]. By incorporating this algorithm, the collision check follows the path, meaning that in the worst-case scenario, the entire path is calculated. However, the overall computational load is reduced because the algorithm avoids unnecessary calculations as soon as a collision is detected. This approach is particularly efficient in mazes or scenarios where walls are numerous and collisions are likely to occur frequently.

#### 2.2.2. Experiments

To validate the performance, a binary grayscale image (map) with a size of 640 × 640 was used. Walls were represented by 0, and open pathways by 1 (=255). The erode function was applied to secure a safe distance between the robot and the walls. The criterion for the erosion iteration is the number of pixels corresponding to one-half of the robot’s size. Suppose the number of erosion iterations is smaller than this criterion. In that case, a collision may still occur during the robot’s actual movement, even if the program execution results indicate no collision between the robot and the wall. In Figure 2c, the green area represents the safe distance secured through erosion.

#### 2.2.3. Performance Evaluation Metrics

For performance evaluation, the following metrics were compared: the time taken for simplification, the total time from path generation to simplification, the total path distance, the total turning angle, the number of pixels included in the path, and the number of pixels colliding with the wall.

To verify collisions with the wall, a simplified path was drawn on the map with a thickness of 1, using the applied safe distance, and the number of overlapping pixels was quantified numerically. Since the robot’s linear and angular velocities are constant, a smaller total distance and total angle will result in a shorter navigation time for the robot. Furthermore, only when the number of pixels colliding with the wall is zero is the safe distance secured along the entire path.

## 3. Results & Discussion

**Figure 3:**
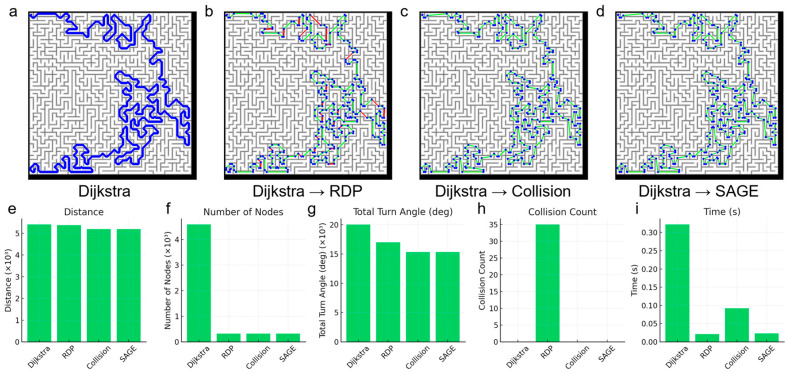
Quantitative evaluation of the in a maze-shaped environment. Representative paths produced by (**a**) Dijkstra (baseline), (**b**) Dijkstra → RDP, (**c**) Dijkstra → Collision-only elimination (checks all possible line shortcuts) and (**d**) Dijkstra → SAGE. In the graph, blue dots represent nodes, green lines represent paths without collision and red lines represent paths with collision. Quantitative comparisons are shown for (**e**) total path length, (**f**) number of nodes, (**g**) cumulative turning angle, (**h**) collision count and (**i**) algorithm runtime. RDP reduces nodes/turns but frequently collides with walls, whereas SAGE achieves RDP-like simplification while preserving a collision-free path at lower computational cost than collision-only elimination.

Figure 3 shows the generated path, where blue dots denote the constituent pixels and green solid lines represent the straight segments connecting them. Travel distance and rotation information for the robot are derived from the pixel information of the blue dots. Crucially, a small number of blue dots leads to a reduction in the required control data and contributes to an increase in the robot’s driving speed due to the consolidation of travel segments.

The path generated by the Dijkstra algorithm stores every constituent pixel as path information, which results in the path appearing as a solid blue line, as shown in Figure 3a. Furthermore, this path includes unnecessary path segments. Segments that involve a sharp turn after moving in a straight line not only increase the travel distance but also contain unnecessary rotations; therefore, such segments must be removed.

To compare the performance of path simplification algorithms, we utilized the RDP algorithm, the Collision-Free algorithm, and the proposed SAGE algorithm in this paper. The Collision-Free algorithm simplifies the path for all pixels without using angular information, then checks if the resulting simplification causes a collision.

The path simplification result using the RDP algorithm with a tolerance of 1 is shown in Figure 3b. In the figure, the red solid line indicates the path segments that resulted in a collision with the wall. Neither the Collision-Free algorithm nor the SAGE algorithm caused any collisions. This successful outcome can also be confirmed in Figure 3h. When comparing the three algorithms, the Collision-Free algorithm exhibited the longest simplification time, while the SAGE algorithm demonstrated a speed similar to that of the RDP algorithm. Referring to the results in Figure 3e–i, the SAGE algorithm confirms its superior simplification performance compared to RDP while maintaining a similar level of simplification speed.

**Figure 4:**
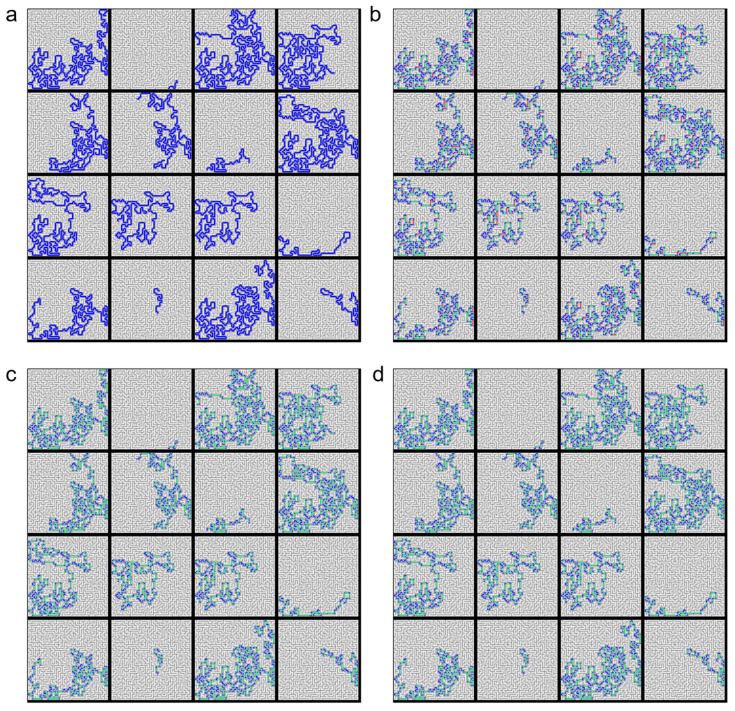
Statistical performance evaluation across various start–goal pairs. Paths of 16 different randomly selected start–goal pairs produced by (**a**) Dijkstra (baseline), (**b**) Dijkstra → RDP, (**c**) Dijkstra → Collision-only elimination (checks all possible line shortcuts) and (**d**) Dijkstra → SAGE. In the graph, blue dots represent nodes, green lines represent paths without collision and red lines represent paths with collision.

**Figure 5:**
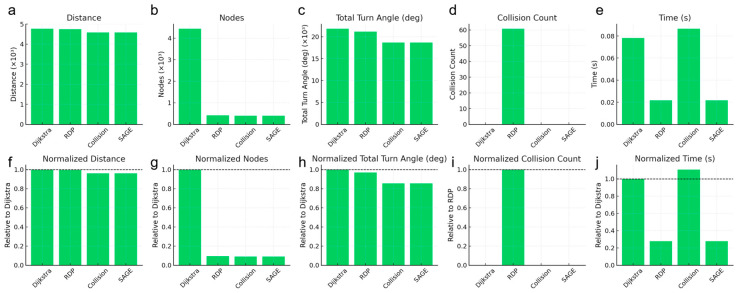
Quantitative evaluation corresponding to the paths shown in Figure 4. Statistical results of (**a**) total distance, (**b**) number of nodes, (**c**) cumulative turning angle, (**d**) collision count and (**e**) computation time among paths produced by Dijkstra (baseline), Dijkstra → RDP, Dijkstra → Collision-only elimination and Dijkstra → SAGE. Normalized comparisons of (**f**) distance, (**g**) number of nodes, (**h**) cumulative turning angle, (**i**) collision count and (**j**) computation time are presented relative to the Dijkstra baseline. The results show that while RDP reduces the number of nodes and turning angles, it causes frequent wall collisions. The Collision-only method eliminates collisions but requires longer computation time. In contrast, SAGE maintains complete collision avoidance while achieving shorter path length, lower turning cost and comparable computational efficiency.

To compare the performance of the algorithms across various environments, we established 16 different scenarios with randomly set start and end points within a complex maze, as shown in Figure 4 and Figure 5. The results confirmed that both the Collision-Free algorithm and the SAGE algorithm successfully simplified the paths without any collisions.

**Figure 6:**
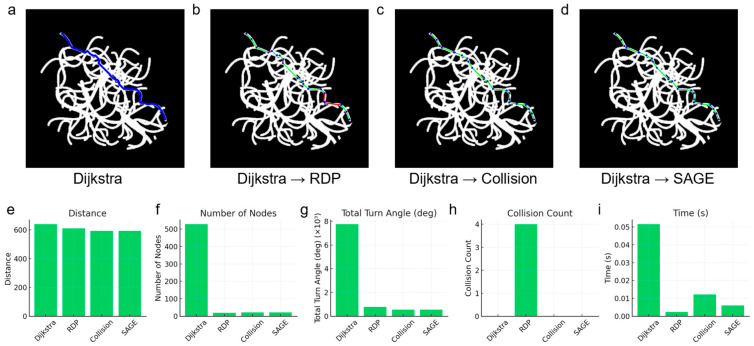
Scalability of the SAGE algorithm in a vascular-shaped environment. Representative paths produced by (**a**) Dijkstra (baseline), (**b**) Dijkstra → RDP, (**c**) Dijkstra → Collision-only elimination (checks all possible line shortcuts) and (**d**) Dijkstra → SAGE. In the graph, blue dots represent nodes, green lines represent paths without collision and red lines represent paths with collision. Quantitative comparisons of (**e**) total path distance, (**f**) number of nodes, (**g**) cumulative turning angle, (**h**) collision count and (**i**) computation time among the same methods.

To simulate the navigation of a microrobot within a blood vessel, an experiment was conducted using a virtual vascular model (Figure 6). Due to the potentially fatal risk to the patient if the vessel is damaged, collision avoidance is of paramount importance in this scenario. The path generated by the Dijkstra algorithm showed no collisions; however, it contained too many nodes, resulting in a long total distance and a large total rotation angle. The RDP simplification reduced the total distance, total rotation angle, and total number of nodes, but a collision occurred at approximately the two-thirds point of the path. The Collision-Free algorithm successfully simplified the path without collision, though the time required for simplification was considerable. In contrast, the simplification results using the SAGE algorithm showed a shorter total distance and a smaller total rotation angle compared to RDP. Furthermore, the SAGE algorithm achieved simplification without collision and demonstrated a faster simplification speed than the Collision-Free algorithm.

**Figure 7:**
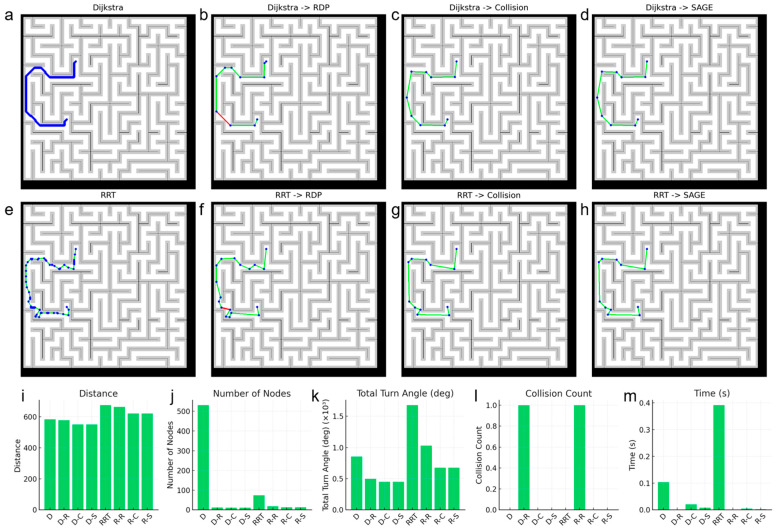
Scalability of the SAGE algorithm combined with different path planners (Dijkstra or RRT). Representative paths produced by (**a**) Dijkstra (baseline), (**b**) Dijkstra → RDP, (**c**) Dijkstra → Collision-only elimination and (**d**) Dijkstra → SAGE. Representative paths produced by (**e**) RRT (baseline), (**f**) RRT → RDP, (**g**) RRT → Collision-only elimination and (**h**) RRT → SAGE. In the graph, blue dots represent nodes, green lines represent paths without collision and red lines represent paths with collision. Quantitative comparisons of (**i**) total path distance, (**j**) number of nodes, (**k**) cumulative turning angle, (**l**) collision count and (**m**) computation time among all tested methods.

Although the Dijkstra algorithm exhibits superior performance to the RRT algorithm in 2D environments, the RRT algorithm is more commonly utilized in 3D environments. Therefore, we verified whether the proposed algorithm is also suitable for use in conjunction with the RRT algorithm (Figure 7). Since the RRT algorithm generates a path by randomly selecting the next node, the pixels on the path are not necessarily contiguous, resulting in a smaller number of nodes compared to the Dijkstra algorithm. As shown in Figure 7b,f,l, when the paths generated by the two path generation algorithms were simplified using the RDP algorithm, collisions occurred in both cases. In contrast, both the Collision-Free algorithm and the SAGE algorithm successfully avoided collisions. Furthermore, when simplification was performed using the SAGE algorithm, both the total path distance and the total rotation angle were smaller in both scenarios compared to when using the RDP algorithm. The simplification speed of the SAGE algorithm was also faster than that of the Collision-Free algorithm.

**Figure 8:**
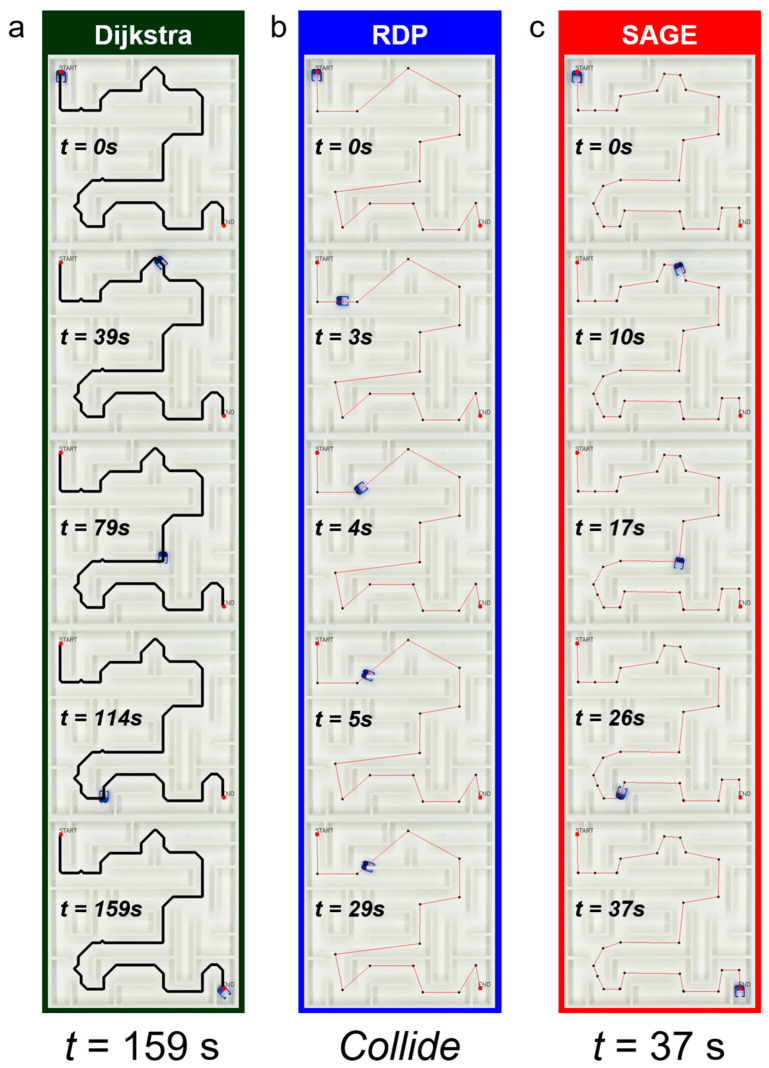
Microrobot navigation in a physical maze. Time-lapse sequences show (**a**) Dijkstra completing the route (≈159 s), (**b**) Dijkstra → RDP colliding and failing to finish and (**c**) Dijkstra → SAGE completing collision-free in ≈37 s. The SAGE-simplified path substantially shortens travel time without collision.

We validated the robot’s actual operation in a real-world maze based on paths generated using the Dijkstra algorithm, Dijkstra with RDP, and Dijkstra with SAGE (Figure 8 and Supplementary Videos S1–S3). When only the Dijkstra algorithm was used, the robot successfully moved from the start point to the goal point without collision, but it took an average of 158.6 s to complete the navigation (Figure 8a). When the path was simplified using the RDP algorithm, a collision occurred, causing the robot to become stuck against the wall and unable to move further (Figure 8b). However, the path generated using the SAGE algorithm allowed the robot to follow it successfully without collision, significantly reducing the travel time to an average of 37 s (Figure 8c).

**Figure 9:**
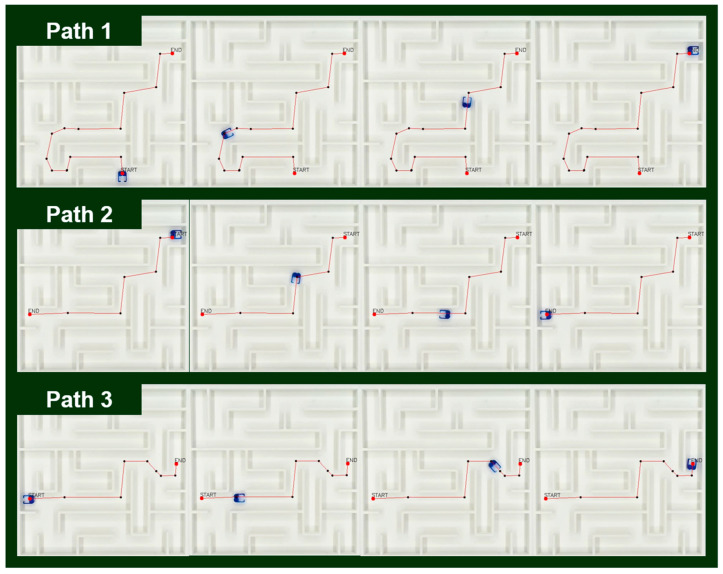
Multi-path task demonstration with SAGE. The microrobot executes three sequential virtual missions in a maze. (**Path 1**) Autonomous travel to a pickup site, (**Path 2**) transport from pickup to a designated location and (**Path 3**) return to the final destination. All missions are completed without collision, demonstrating the reliability, reusability, and autonomous task-planning capability of the *SAGE* algorithm for continuous microrobot operation.

Next, we demonstrated the system’s ability to handle dynamic task-chaining by receiving sequential new endpoints in real time (Figure 9 and Supplementary Video S4). When a new endpoint is provided (e.g., via a mouse callback), a new path is generated using the Dijkstra algorithm only from the robot’s current location (which was the previous endpoint) to the new target. This newly generated path segment is then immediately simplified by the SAGE algorithm and appended to the robot’s command queue. This incremental approach avoids the computational overhead of recalculating the entire multi-segment journey from the original starting point.

In the experiment depicted in Figure 9, the robot received a total of three sequential endpoints. In all three cases, the system successfully generated and executed a new, safe path from the robot’s current position to the next target without collision, confirming its ability to reliably update navigation tasks in real time. This rapid incremental simplification capability demonstrates that SAGE can be readily applied to interactive automation systems or environments with real-time task changes. This result demonstrates the ability of the SAGE algorithm to adapt to sequential goal updates.

## 4. Limitations & Future Development

Although the proposed Secure Angle-based Geometric Elimination (SAGE) framework demonstrates effective geometric path simplification and sequential goal adaptation, the present validation was performed under quasi-static conditions. In the current setup, the entire path is generated and simplified before actuation and each motion segment is executed independently. This pre-planned operation ensures deterministic reproducibility but restricts responsiveness to environmental variations, moving obstacles, or fluidic perturbations that frequently occur in real microrobotic environments. A primary limitation arises from the absence of continuous sensory feedback. The present 2D linear-stage platform executes motions open-loop, without feedback during movement. Consequently, the system cannot compensate for drift, vibration or external flow disturbances. In addition, actuation updates and path computation occur asynchronously, introducing latency between planning and execution. These constraints limit the dynamic adaptability of the SAGE framework when applied to fluidic or vascular-like environments.

Future developments will focus on transforming SAGE from a quasi-static planner into a real-time adaptive navigation framework. Specifically, visual tracking will be integrated with incremental SAGE updates to enable step-wise replanning synchronized with microrobot movement. Furthermore, we plan to extend the SAGE framework toward active droplet manipulation and lab-on-a-chip automation, where microrobots can actively transport, merge and split droplets within microfluidic channels under closed-loop visual and magnetic control. This direction will bridge path planning with functional micromanipulation, allowing on-chip operations such as reagent mixing, localized biochemical reactions and programmable fluid routing. These advancements will ultimately enable the SAGE framework to evolve into a fully dynamic, feedback-driven microrobot navigation and manipulation platform suitable for adaptive microfluidic automation and in-vitro biomedical experimentation.

## 5. Conclusions

We presented the Secure Angle-based Geometric Elimination (SAGE) algorithm, a single-pass path-simplification method designed for real-time microrobot navigation in constrained environments. By combining angle-based filtering with collision checking, SAGE converts pixel-level shortest paths into low-complexity, collision-free trajectories. Quantitative evaluations demonstrated that SAGE achieved approximately 5% shorter path length, 20% lower cumulative turning cost, and 0% collision rate while maintaining computation time comparable to the Ramer–Douglas–Peucker algorithm. Scalability tests across maze-like and vascular-shaped environments confirmed robust performance with both Dijkstra and RRT planners. Physical microrobot experiments verified complete collision-free motion and more than 75% reduction in navigation time compared with unsimplified paths. Multi-path tasking further demonstrated autonomous, continuous operation through successive pickup, transport, and return missions. These results establish SAGE as a reliable, efficient, and scalable path-simplification framework for future lab-on-a-chip, chemical-reaction, and molecular-diagnostic microrobot automation systems.

## Data Availability

The original contributions presented in this study are included in the article/Supplementary Material. Further inquiries can be directed to the corresponding authors.

## Supplementary Materials

The following supporting information can be downloaded at: https://www.mdpi.com/article/10.3390/mi16111273/s1, Video S1: Microrobot path planning using Dijkstra., Video S2: Microrobot path planning using RDP., Video S3: Microrobot path planning using SAGE., Video S4: Multi-path task demonstration using SAGE.
